# Reducing Inhaler Waste and Costs Through Sustainable Interventions

**DOI:** 10.1016/j.chest.2025.08.012

**Published:** 2026-02-09

**Authors:** Marianne Laguë, Isabelle Giroux, Alexandre Sanctuaire, Julie Racicot, Cassiopée Gagnon-Paradis, François Maltais, Andréanne Côté, Krystelle Godbout

**Affiliations:** a Faculty of Medicine, Université Laval, Quebec, QC, Canada; b Institut universitaire de cardiologie et de pneumologie de Québec, Université Laval, Quebec, QC, Canada

In Canada, the health care system accounts for 4.6% of greenhouse gas (GHG) emissions, 25% of which originate from medication use.^1^ Metered-dose inhalers (MDIs) are of particular concern as hydrofluoroalkane, the propellant within the device, is a potent GHG, retaining warmth 1,300 times more than CO_2_.^1^ With the GHG emissions of 1 inhaler being equivalent to a 170-km car drive, MDIs alone generate 13 billion tons of CO_2_ equivalent annually.^1^ A recent Canadian Thoracic Society Position Statement emphasizes the prescriber’s role in reducing the inhaler-related carbon footprint by promoting dry-powder inhalers (DPIs).^2^ This can be achieved through increased awareness while maintaining shared decision-making. However, individual level interventions have limited impact, and systematic interventions are required to reduce inhaler-related GHG emissions. Hospitals are major emission sources, which makes them key targets for GHG reduction efforts. Creating a Sustainable Canadian Health System in a Climate Crisis (CASCADES) is a pioneer initiative that supports the Canadian health-care system in an ecologic transition toward a more sustainable system.^1^^,^^2^ They produce individual and hospital-focused resources to assist establishments in their transition.^1^^,^^2^

The Institut Universitaire de Cardiologie et de Pneumologie de Québec is a specialized center in respiratory medicine, and this mission results in a high consumption of inhalers. A local initiative was instigated by pharmacists and respiratory therapists to quantify the burden of inhaler-related GHG emissions and improve inhaler management along the respiratory trajectory.^3^ A multidisciplinary taskforce was created subsequently to address challenges in reaching these objectives. Four critical areas for improvement were identified: the pulmonary function laboratory (PFL), the emergency department, the hospitalization ward, and the hospital pharmacy.

In the PFL, it was observed that a new MDI with a disposable spacer was used for every patient whose condition required bronchodilator administration, for a total of 93 inhalers and spacers per week. The inhalers were discarded after each use, which resulted in significant medication waste. A disinfection procedure was developed and implemented in collaboration with the hospital infection prevention and control service that allowed reuse of MDIs between patients. A manual log is maintained with each inhaler to track the number of doses delivered; the inhaler is discarded after all doses have been administered. The disposable spacers were also replaced with reusable unidirectional valve holding chambers that can be sterilized up to 100 times. These changes led to a 97% reduction in MDI consumption from 93 inhalers per week to only 3, which decreased GHG emissions by 136,000 kg of CO_2_ equivalent. Annually, reuse of the inhalers reduced costs by 18,000 Canadian dollars, and the replacement of disposable spacers is predicted to save an additional 10,000 Canadian dollars.

A similar issue involving single-patient use of bronchodilator MDIs was found and addressed in the emergency department. A disinfection procedure like the one used in the PFL was implemented. However, keeping track of the doses delivered by each inhaler was deemed difficult to achieve. To mitigate this issue, the MDIs are weighed regularly and discarded when they reach their “empty weight.” The financial and environmental impact of this measure in the emergency department is not yet known, but the results can be expected to be similar to those in the PFL, because their annual bronchodilator budgets align. Another source of medication waste that was identified in the emergency department was the supply of maintenance inhalers for short stays that did not result in hospitalization. Because of legal considerations, patients were not allowed to take home the hospital-provided inhalers that were discarded at discharge nor were they allowed to take their own medication. After obtaining appropriate approval, using the patient's own maintenance inhalers is now allowed in the emergency department, which offers further opportunities to reduce inhaler waste.

Moving hospitalized patients to another room or ward was identified as a significant inhaler waste source and misplacement, loss, or duplication of inhalers frequently occurred on the hospital ward. To prevent duplication of inhalers supplied by the hospital pharmacy that ended up being discarded, all inhalers are now kept on wards in dedicated locked carts and are supplied by respiratory therapists; inhalers that are used in the emergency department now systematically follow the corresponding patients to the ward. This prevents having to provide multiple inhalers of the same medication to the same patient. To further decrease the carbon footprint, automatic substitutions from MDIs to DPIs were implemented by the respiratory therapists when clinically appropriate. Inspiratory flows are assessed systematically before substitution when risk factors for low inspiratory flows are present.^4^, ^5^, ^6^

Actions were also taken in the hospital pharmacy to decrease our hospital carbon footprint and medication waste. Because equivalent MDIs marketed by different companies have different carbon footprints, when possible, lower carbon footprint options (including smaller hospital format inhalers) were prioritized for use.^7^ Also, cost savings associated with the implemented measures were used to purchase specialized collection boxes to recycle the inhalers used in the hospital.

Future steps are already in development. They include MDIs replacement by nebulization for patients in the ICU who are intubated^8^ and automatic continuation at discharge of MDI to DPI substitution when this was made during the hospitalization. We are also looking for ways to legally allow patients to take home hospital-provided inhalers.

Systematic changes in the way inhalers are used in our hospital lead to a substantial drop in MDI waste with consequent reduction in GHG emission and health care savings (Fig 1). Our local experience emphasizes the potential impact of large-scale interventions to fight climate change. In addition to individual efforts to lower prescription-related carbon footprints, hospitals must adopt a structured, institution-wide approach to sustainability by integrating carbon-conscious decisions in all levels of clinical and administrative practice. Additionally, important cost reduction happened by reducing unnecessary inhaler delivery, minimizing medication waste, replacing disposable devices, and replacing MDIs for DPIs when possible. Clinicians, hospital administrators, and government can and must advocate for sustainable prescriptions and hospital-wide carbon reduction strategies that should be embedded in standard practice to ensure that climate-conscious health care is both financially viable and clinically effective.

**Figure 1:**
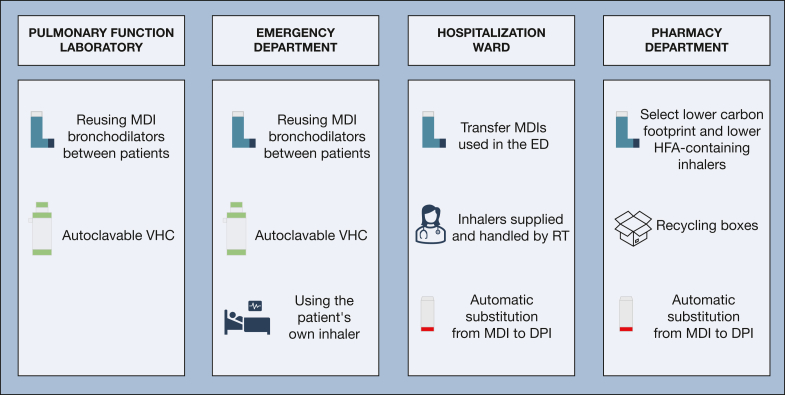
Systematic changes in inpatient inhaler management in 4 critical areas at the Institut Universitaire de Cardiologie et de Pneumologie de Québec. DPI = dry powder inhaler; ED = emergency department; HFA = hydrofluoroalkane; MDI = metered-dose inhaler; RT = respiratory therapist; VHC = valve holding chamber.

## Financial/Nonfinancial Disclosures

The authors have reported to *CHEST* the following: K. G. declares the following: Consultancy/Advisory Board for Astra Zeneca, Covis, GlaxoSmithKline, Roche, Sanofi, TEVA, Valeo; Speaker Honoraria for Astra Zeneca, Boehringer, Covis, GlaxoSmithKline, Novartis, Valeo; Funded Grants or Clinical Trials by Astra Zeneca, Bellus, GlaxoSmithKline, Regeneron, Roche, Sanofi, Valeo, Viatris. A. C. declares the following: Consultancy/Advisory Board for Astra Zeneca, GlaxoSmithKline, Sanofi, Regeneron; Speaker Honoraria for Astra Zeneca, GlaxoSmithKline, Sanofi, Regeneron; Funded Grants or Clinical Trials by Astra Zeneca, GlaxoSmithKline. F. M. declares the following: Consultancy/Advisory Board for Roche; Speaker Honoraria for GlaxoSmithKline; Funded grants or clinical trials by Astra Zeneca, GlaxoSmithKline, Grifols, Novartis, Sanofi; Receipt of equipment, material by Oxynov. None declared (M. L., I. G., A. S., J. R., C. G.-P.).

## References

[1] Stoynova V., Culley C. Climate conscious inhaler practices in inpatient care version 0 (2023) [Internet]. CASCADES (Creating a Sustainable Canadian Health System in a Climate Crisis). CASCADES Canada website. https://cascadescanada.ca/resources/climate-conscious-inhaler-practices-in-inpatient-care-playbook/.

[2] Gupta S., Couillard S., Digby G. (2023). Canadian Thoracic Society position statement on climate change and choice of inhalers for patients with respiratory disease. Can J Respir Crit Care Sleep Med.

[3] Giroux I., Paradis-Gagnon C., Denis C. (2025). Sélection et utilisation écoresponsable des médicaments administrés par inhalation à l’Institut universitaire de cardiologie et de pneumologie de Québec – Université Laval. Pharmactuel.

[4] Côté A., Beach J., Reynolds J. (2024). OCS use in uncontrolled severe asthma in Canada [Conference abstract]. Eur Respir J.

[5] Janssens W., VandenBrande P., Hardeman E. (2008). Inspiratory flow rates at different levels of resistance in elderly COPD patients. Eur Respir J.

[6] Ghosh S., Ohar J.A., Drummond M.B. (2017). Peak inspiratory flow rate in chronic obstructive pulmonary disease: implications for dry powder inhalers. J Aerosol Med Pulmon Drug Deliv.

[7] Stoynova V., Culley C. Detailed inhaler comparison chart (2023) [Internet]. CASCADES (Creating a Sustainable Canadian Health System in a Climate Crisis). CASCADES Canada website. https://view.publitas.com/5231e51e-4654-42c2-accd-b722e21f3093/detailed-inhaler-comparison-chart-preview/page/1?_gl=1*fnkti3*_ga*MTcyNDUyNTQ0MC4xNzQ1OTIxNzIw*_ga_TRM5NF4JFC*MTc0NTkyMTcxOS4xLjEuMTc0NTkyMTc2NS4wLjAuMA.

[8] Li J., Liu K., Lyu S. (2023). Aerosol therapy in adult critically ill patients: a consensus statement regarding aerosol administration strategies during various modes of respiratory support. Ann Intensive Care.

